# The Transcriptional Kinases CDK8/19 in the Regulation of the Macrophage Inflammatory Response

**DOI:** 10.32607/actanaturae.27815

**Published:** 2026

**Authors:** A. N. Neznamov, Yu. A. Oparina, D. M. Dolmatova, L. A. Ilchuk, M. V. Kubekina

**Affiliations:** Russian Academy of Science, Institute of Gene Biology, Moscow, 119334 Russia; Russian Academy of Science, Engelhardt Institute of Molecular Biology, Moscow, 119334 Russia

**Keywords:** macrophages, functional characteristics of macrophages, inflammation, transcriptional kinases CDK8 and CDK19

## Abstract

Macrophage dysfunction is a key pathogenetic mechanism in the progression of a wide range of human chronic inflammation conditions, including atherosclerosis, rheumatoid arthritis, and metabolic disorders. The CDK8 and CDK19 paralogous kinases, the subunits of the Mediator complex acting as transcription regulators, are essential modulators of the inflammatory response. This study addresses the role of CDK8/19 kinases in the macrophage inflammatory response via multiple mechanisms: activation of pro- and anti-inflammatory genes and surface markers, STAT1 pathway modulation, activation of glycolytic cascade genes, regulation of the dynamics of lipid inclusions, and phagocytic activity. Experiments have shown that CDK8 and CDK19 may exhibit functional divergence. Cdk19 knockout reveals that CDK19 plays a role in the suppression of the M1 response, as well as the regulation of lipid homeostasis and phagocytosis. Double Cdk8/19 knockout macrophages are characterized by an exacerbated anti-inflammatory response while preserving normal lipid accumulation and phagocytosis levels. Inhibition of CDK8/19 kinase activity reproduces these effects only partially, suggesting that both kinase-dependent and kinase-independent mechanisms of action exist. The identified effects reveal previously unknown regulation mechanisms of macrophage immunometabolism. Modulation of CDK8 and CDK19 activity can become a novel therapeutic target for a wide range of chronic inflammatory conditions and metabolic disorders associated with macrophage dysfunction.

## INTRODUCTION

Chronic inflammatory disorders are currently among the major concerns in medicine, imposing a significant burden on the healthcare system in both developed and developing countries [1, 2]. Atherosclerosis, rheumatoid arthritis, and metabolic disorders are examples of such conditions [3, 4, 5, 6].

Macrophages play a crucial role in the pathogenesis of inflammatory diseases, since they can dynamically switch their phenotype in response to microenvironmental signals [3, 7]. The proinflammatory M1 and anti-inflammatory M2 macrophagic states have been traditional distinctions [8]. M1 macrophages are activated by interferon gamma (IFN-γ) and lipopolysaccharides (LPS); they predominate in chronic inflammation sites. Their formation is dependent on activation of the NF-κB transcription factor. NF-κB induces expression of pro-inflammatory genes (Tnf, IL-1β, IL-6, and IL-12) and STAT1, which is required for Nos2 expression and nitrogen oxide production [9, 10, 11]. C/EBPβ cooperates with NF-κB to enhance transcription [12]. The joint action of these factors leads to the classical M1 phenotype, which is characterized by the secretion of proinflammatory cytokines and reactive oxygen species, resulting in tissue destruction and promoting further inflammation [3, 4, 6, 7, 8]. In contrast, M2 macrophages are induced by IL-4 and IL-13 via transcription factor activation by STAT6 [10]. Other factors, such as PPARγ/δ and KLF4, also affect the development of the M2 phenotype [13]. It facilitates expression of M2- specific genes (ARG1 and MRC1); arginine metabolism shifts toward polyamine production and tissue repair [14, 15]. Secretion of anti-inflammatory cytokines (IL-10 and TGF-β) is enhanced [7, 8]. The transition from acute to chronic inflammation is accompanied by macrophage dysfunction; the normal balance shifts toward the M1 phenotype [6, 7, 16, 17, 18], making M1 macrophages a promising therapeutic target.

Macrophage polarization is tightly linked to their metabolic reprogramming (M1 rely on glycolysis, while M2 rely on oxidative phosphorylation) [4, 19] and is strictly controlled transcriptionally. Cyclin-dependent kinase CDK8 and its paralog CDK19 are the key transcriptional regulators [20, 21]. These kinases are components of the Mediator kinase module, which acts as a major link between transcription factors and RNA polymerase II, thus regulating transcription initiation [20, 21]. CDK8/19 modulate the activity of both the whole Mediator complex and the key transcription factors that modulate the inflammatory response (such as NF-κB and C/EBPβ [12]), phosphorylate STAT1 at Ser727, enhancing its transcriptional activity in response to IFN-γ [22, 23], and participate in epigenetic remodeling and splicing control [12, 22]. CDK8/19 dysregulation is observed in cancer [24, 25]. Their inhibition suppresses the expression of proinflammatory genes and shows a therapeutic potential in models of autoimmune diseases [26, 27].

Considering the pivotal role of macrophages in the initiation and sustenance of chronic inflammation, as well as the growing body of data suggesting involvement of transcriptional kinases CDK8 and CDK19 in the immune response regulation [8, 28, 29, 30], we studied the effect of CDK8/19 on the development of an inflammatory response in M1-polarized macrophages. This study unveils novel kinase-independent functions of these proteins in macrophages. We have for the first time shown that CDK19 exhibits a STAT1-independent suppressive activity against M1 inflammation. Cdk19 knockout results in hyperactivation of IL-1β and Nos2, as well as reduces phagocytosis and lipid accumulation. Interestingly, double Cdk8/19 knockout is associated with the activation of anti-inflammatory mechanisms. Selective inhibition of CDK8/19 kinase activities does not fully reproduce the genetic knockout phenotype. The collected data paint complex non-additive interactions between CDK8 and CDK19. These findings prove that these proteins possess both kinase-dependent (e.g., IL-10 activation) and kinase-independent functions (reduced activation of IL-1β and Tnf).

## EXPERIMENTAL

**The mouse strains used in the study**

The Rosa26/Cre-ERT2 mouse strain was used in this work as a control. The experimental group comprised the Rosa26/Cre-ERT2 mouse strain, with constitutive Cdk19 knockout and loxP-flanked exon 2 of the Cdk8 gene (Rosa26/Cre-ERT2/Cdk8fl/fl/Cdk19−/−). These mice were produced by cross-breeding of three mouse lines, Cdk19−/− (RRID:MMRRC_047035-UCD), obtained from the Mutant Mouse Resource & Research Center, Cdk8fl/fl (Jax:008463), and Rosa26Cre-ERT2 (B6.129-Gt(ROSA)26Sortm1(cre/ERT2)Tyj/J), obtained from the Jackson Laboratory. Offspring genotyping was performed according to the previously published protocol [31].

The mice were kept in the animal facility of the Core Facility Center, Institute of Gene Biology of the Russian Academy of Sciences, with ad libitum access to food and water. The animals were housed on a 12-h on/off light cycle; air temperature was kept at 23 ± 1°C; and humidity, at 42 ±  5%. All the experimental procedures involving the laboratory animals were approved by the Bioethics Committee of the Institute of Gene Biology, Russian Academy of Sciences.

**Primary culture of bone marrowderived macrophages**

Macrophages were derived from mouse bone marrow as per the standard protocol [32]. The animals were put down by cervical dislocation; the femurs and tibias were dissected out with intact epiphysis under sterile conditions and scraped of muscle tissue. A syringe with a 25G needle was filled with 5 mL of ice-cold DMEM (Gibco, USA) supplemented with 20% FBS (Cytiva, USA), 50 U/mL penicillin and 50 µg/mL streptomycin. Epiphyses were aseptically removed with sterile scissors; a 25G needle was inserted into the medullary cavity; and bone marrow cells were flushed out. The collected cell suspension was thoroughly mixed; cell concentration was calculated using a hemocytometer (Minimed, Russia) and diluted with fresh media to a cell density of 106 /mL. Cells were cultured at 37°C, 5% CO_2_ , for 7 days in the presence of a 10 ng/mL macrophage colony-stimulating factor M-CSF (SciStore, Russia). Pro-inflammatory induction was performed in vitro on day 7 by addiction of 100 ng/mL LPS (Sigma-Aldrich, USA) and 20 ng/mL IFN-γ (Peprotech, USA).

**Cdk8 gene knockout induction and CDK8/19 kinase inhibition in a primary macrophage culture**

Cdk8 gene knockout was induced under standard culture conditions in vitro on day 2 by addition of 2 µM 4-hydroxytamoxifen (4-OHT, Sigma-Aldrich, USA) to culture media for 120 h. The selective CDK8/19 inhibitor senexin B (Biocad, Russia), at a concentration of 1 µM, was used for kinase activity suppression 4 h prior to proinflammatory stimulation.

**Experimental design**

The following experimental groups were used in the study:

Rosa − macrophages from the Rosa26/Cre-ERT2 mouse strain.

Senexin B − macrophages from the Rosa26/Cre-ERT2 mouse strain treated with Senexin B with knockeddown CDK8/19 kinase activity.

CDK19 − macrophages from the Rosa26/Cre-ERT2/ Cdk8fl/fl/Cdk19−/− mouse strain with constitutive Cdk19 gene knockout.

CDK8/19 − macrophages from the Rosa26/Cre-ERT2/ Cdk8fl/fl/Cdk19−/− mouse strain with double Cdk8/19 knockout treated with 4-OHT.

Tamoxifen − macrophages from the Rosa26/Cre-ERT2 mouse strain treated with 4-OHT.

**Gene expression analysis**

RNA was extracted from the samples using the ExtractRNA reagent (Evrogen, Russia), according to the manufacturer’s protocol. Complementary DNA was synthesized using the MMLV RT kit (Evrogen, Russia), according to the manufacturer’s protocol. Amplification was achieved using the qPCRmix-HS master mix with a SYBR intercalating dye (Evrogen, Russia) and primers (Supplementary Table S1). Relative gene expression was analyzed by quantitative real-time PCR. Fold changes were calculated using the ΔΔCt method; Rosa group was used as a control; the Hprt1 mRNA levels were used for normalization.

**Surface marker analysis**

Production of macrophage surface markers was assessed by fluorescence flow cytometry on a CytoFLEX analyzer (Beckman Coulter, USA). Based on published data, the following antibodies were used: FITC-labeled anti-CD14 antibody (Biolegend, USA) – a monocyte and macrophage marker; Pacific Blue anti-CD86 antibody (Biolegend, USA) – an M1 macrophage marker. The proportion of double-positive CD14^+^CD86^+^ cells was measured. Macrophages were washed off the plastic using a Versene solution (Bioinnlabs, Russia) prior to staining. Unstained controls were used to avoid possible autofluorescence effects and ensure correct target population identification. At least 30,000 events per sample were detected to characterize macrophage populations.

**Analysis of lipid inclusions**

Analysis of the lipid contents was performed 48 h post proinflammatory stimulation using the LipidSpot 488 live cell stain (Biotium, USA). Nuclei were stained with Hoechst 33342 (Sigma-Aldrich, USA). The cells were visualized using an ECLIPSE Ti fluorescent microscope (Nikon, Japan); at least 10 fields of view were captured per well. Image analysis and measurements were performed using the ImageJ software. The integral LipidSpot signal intensity (excluding background autofluorescence) for each field of view was normalized to the Hoechst 33342 integral intensity to account for cell density variation.

**Analysis of the phagocytic activity of macrophages**

The phagocytic activity of macrophages was measured using fluorescently labeled pHrodo Green E. coli BioParticles (Invitrogen, USA) by fluorescence flow cytometry. The results were additionally corroborated using fluorescent microscopy (ECLIPSE Ti).

**Western blotting**

Samples for analyzing the CDK8 and CDK19 levels were collected after Cdk8 gene knockout induction. CDK8 (D6M3J) rabbit 17395 antibodies (Cell Signaling, USA) and anti-CDK19 antibodies [31] in 1 : 1000 dilution were used for CDK8 and CDK19 detection, respectively. Samples for the STAT1 analysis were collected 6 h post proinflammatory stimulation to detect early phosphorylation events. Stat1 (D1K9Y) rabbit 14994 and Phospho-Stat1 (Ser727) rabbit 8826 (Cell Signaling, USA) antibodies in 1 : 1000 dilution were used for detecting STAT1 and phospho-STAT1, respectively. β-Actin mouse A2228 (Sigma-Aldrich, USA) antibodies in 1 : 1000 dilution were used for loading control. Secondary anti-rabbit IgG–HRP 7074 and anti-mouse IgG–HRP 7076 (Cell Signaling, USA) antibodies were used in 1 : 2000 dilution. Membranes were visualized using the iBright FL1500 system (Invitrogen, USA) and subsequently analyzed using the ImageJ software.

**Statistical analysis**

Statistical analysis was performed by one-way ANOVA using the GraphPad Prism 8.0.1 software (GraphPad Software, USA). At least three biological replicates were used in each experiment; the results are presented as mean ± standard error of the mean.

## RESULTS AND DISCUSSION

**Cdk8/19 and Cdk19 knockouts alter the inflammatory profile of macrophages**

**Fig. 1:**
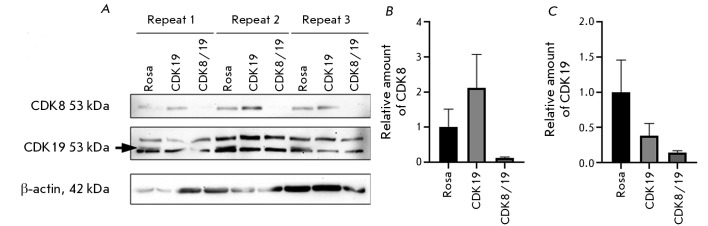
The CDK8 and CDK19 levels in the macrophages of various experimental groups. (A) A representative western blot image. (B) The relative amount of CDK8 normalized to β-actin levels. (C) The relative amount of CDK19 normalized to β-actin levels. Anti-CDK19 antibodies identified extra, non-specific bands; the band of interest is shown with an arrow

Rosa26/Cre-ERT2/Cdk8fl/fl/Cdk19−/− mice and the macrophages extracted from them were used. The macrophages carried a constitutive Cdk19 knockout; Cdk8 knockout was induced by incubation in the presence of 4-OHT. Both the induced Cdk8 knockout and constitutive Cdk19 knockout were confirmed by western blotting (Fig. 1A−C, Supplementary Fig. S1). Despite some dispersion, the diagrams display reduction in the CDK8 and CDK19 protein levels in the Cdk19 and Cdk8/19 knockout lines as compared to the Rosa control, confirming the relevance of our genetic model.

The macrophage inflammatory response is inextricably linked to the regulation of inflammatory gene expression. In order to study the role of CDK8 and CDK19 in the regulation of the macrophage inflammatory response, we first assessed the upregulation levels of the IL-1β, Nos2, Ccl-2, Tnf, Arg1 and IL-10 genes upon M1 polarization. To do this, differentiated macrophages were stimulated with IFN-γ and LPS for 24 h, followed by lysis for RNA extraction and consecutive analysis of the expression levels. Selected genes form a panel that covers the key functional macrophage programs during inflammation and allows one to both determine whether there is inflammation (Tnf) or not, as well as assess which branches of the immune response are affected by CDK8 and CDK19: cytotoxicity (Nos2), ability to recruit immune cells (Ccl-2), pyrogenesis (IL-1β), or inflammation resolution (Arg1 and IL-10) [3, 4, 6, 7, 8].

**Fig. 2:**
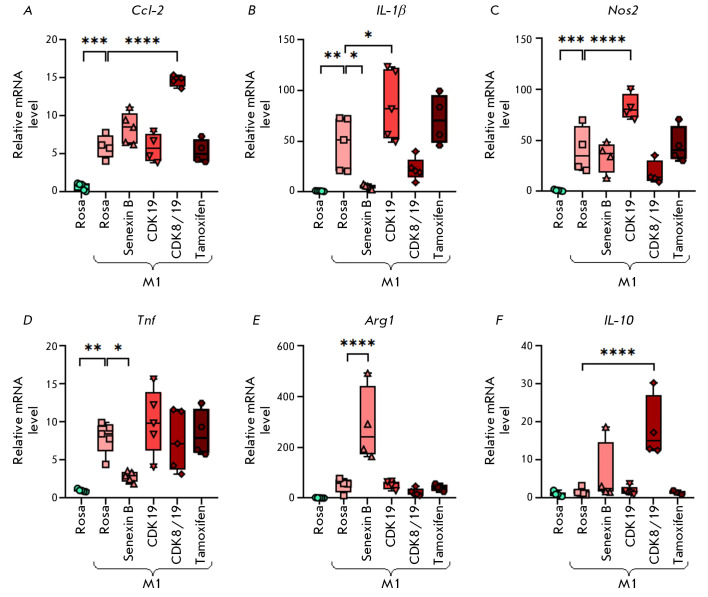
The expression levels of pro- (A–D) and anti-inflammatory (E, F) macrophage responses. The expression levels in the untreated control Rosa group were set to one for each gene analyzed. The values for stimulated (M1) macrophages from experimental groups were compared to those obtained for stimulated Rosa macrophages (*p < 0.05; **p < 0.01; ***p < 0.001; ****p < 0.0001)

Using mouse bone marrow-derived macrophages from Cdk19 knockout and Cdk8/19 double knockout animals, we identified the multidirectional effects of CDK8 and CDK19 on the expression of inflammatory response genes (Fig. 2A−F). Activation of the proinflammatory genes IL-1β and Nos2 was observed in the Cdk19 knockout cells; in the Cdk8/19 double knockout group, their levels were unchanged but Ccl-2 was increased (Fig. 2A−C), suggestive of the role of CDK8/19 in suppressing the ability of macrophages to recruit immune cells. Inhibition of CDK8/19 kinase activity reduced Tnf and IL-1β activation, while genetic knockout had no effect (Fig. 2D). This suggests that non-kinase functions have the potential to play a role in the initiation and maintenance of the inflammatory cascade. Double knockout and inhibition of CDK8/19 kinase activities was accompanied by activation of the anti-inflammatory genes IL-10 and Arg1 (Fig. 2E,F), characteristic of the M2 phenotype [7, 8]. Arg1 activation was only observed in Senexin B group macrophages, suggesting that regulation of the expression of this gene is linked to the non-kinase CDK8/19 function. The collected data suggest the involvement of CDK8/19 in the M1 to M2 phenotype switch, with CDK19 likely an inflammatory response suppressor.

**Fig. 3:**
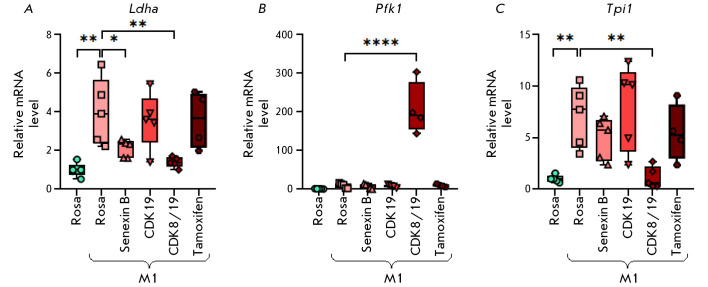
Expression of the glycolytic cascade genes: (A) Ldha; (B) Pfk1; and (C) Tpi1. The expression levels in the untreated control Rosa group were set to one for each gene analyzed. The values for stimulated (M1) macrophages from experimental groups were compared to those obtained for stimulated Rosa macrophages (*p < 0.05; **p < 0.01; ****p < 0.0001)

It is well known that inflammatory M1 macrophages utilize aerobic glycolysis as an energy source, while M2 macrophages rely mostly on oxidative phosphorylation [4, 17]. To assess the role of CDK8/19 in energy metabolism regulation, we analyzed the effect of CDK19 and CDK8/19 deficiency on the expression of the key glycolytic cascade genes, in particular Pfk1 (phosphofructokinase), Ldha (lactate dehydrogenase), and Tpi1 (triosephosphate isomerase) during inflammation stimulation (Fig. 3A−C). Inhibition of CDK8/19 kinase activity reduced Ldha upregulation. Double Cdk8/19 knockout reduced activation of the Ldha and Tpi1 genes and enhanced activation of the Pfk1 gene, a key glycolysis enzyme [34]. Interestingly, Cdk19 knockout had no effect as compared to the control. The observed absence of activation of the Ldha gene encoding the enzyme that catalyzes the final glycolysis step and is critical for the maintenance of the M1 phenotype [4, 19, 35, 36, 37] may suggest a transition to a less inflammatory M2-like condition [38] in the case of CDK8/19 deficiency. Reduced Tpi1 activation in double knockout cells may create a metabolic bottleneck, leading to accumulation of dihydroxyacetone phosphate (DHAP). Accumulated DHAP is potentially redirected to lipid biosynthesis or the non-canonical pathways that promote oxidative stress and affect signal cascades, thus facilitating functional cell reprograming [39, 40, 41, 42, 43].

Hence, we presume that Cdk8/19 knockout, as well as inhibition of CDK8/19 kinase activity, shifts macrophages from the inflammatory M1 phenotype toward the anti-inflammatory M2 phenotype. The observed changes in the expression of glycolysis genes confirm this hypothesis. On the other hand, Cdk19 knockout promotes the development of a “hyperactivated” M1 macrophage state.

**CDK8/19 are not the only regulators of the STAT1 regulatory pathway during macrophage inflammatory induction**

**Fig. 4:**
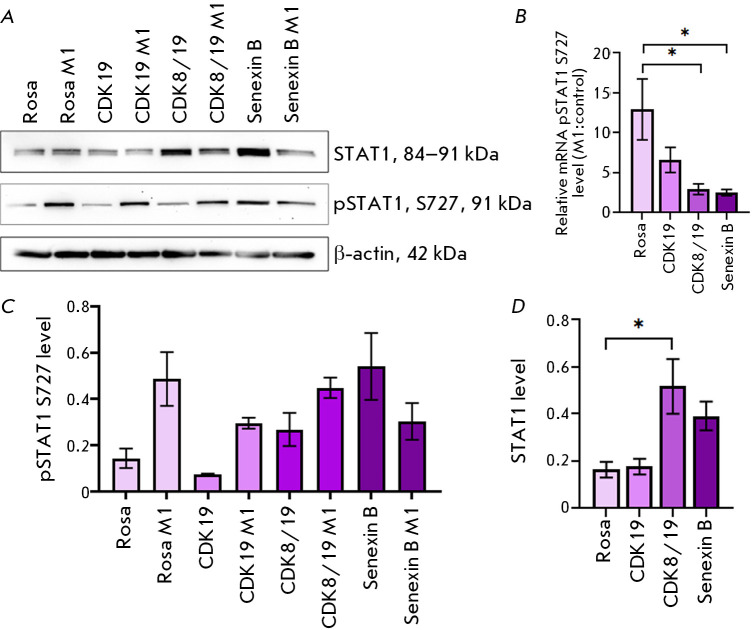
The levels of non-phosphorylated and Ser727-phosphorylated STAT1 in non-stimulated and stimulated (M1) macrophages in the experimental groups. (A) A representative western blot image (one of three replicates). (B) The relative pSTAT1 Ser727 levels normalized to non-phosphorylated STAT1, presented as the ratio between pSTAT1 Ser727 levels in stimulated and non-stimulated macrophages. (C) The phospho-STAT1 levels in stimulated (M1) and non-stimulated macrophages in experimental groups, normalized to β-actin levels. (D) The relative STAT1 levels in non-stimulated macrophages, normalized to β-actin (*p < 0.05)

STAT1 activation via Ser727 phosphorylation is known to be a central event in the macrophage response to IFN-γ, the key M1 polarization inducer. Ser727-phosphorylated STAT1 (pSTAT1(Ser727)) activates the transcription of M1-specific genes [44]. We analyzed the pSTAT1(Ser727) levels to test whether the CDK8 and CDK19 kinases play a role in macrophage phenotype shift. Our findings show that in CDK19 groups, M1 stimulation does not change the STAT1 levels, while the level of its phosphorylation is increased, which was also observed in the control group of Rosa macrophages (Fig. 4A−D, Supplementary Fig. S2). Interestingly, in the CDK8/19 and Senexin B groups, STAT1 levels in non-stimulated macrophages are higher than those in the control Rosa group (Fig. 4A,D) and pSTAT1(Ser727) levels do not change upon M1 induction (Fig. 4A,C). Cdk19 gene knockout does not affect pSTAT1(Ser727) levels as compared to stimulated Rosa macrophages, which initially, we assumed suggested that CDK8 may functionally compensate for its absence. However, the fact that Cdk8/19 double knockout also does not reduce pSTAT1(Ser727) levels upon M1 activation suggests that CDK8 and CDK19 kinase activity is not required for M1 stimuli-induced Ser727 STAT1 phosphorylation. It is possible that this function is assumed by other kinases under double knockout conditions. However, Cdk8/19 knockout macrophages retain their ability to respond to M1 stimuli, since the expression levels of proinflammatory genes are indistinguishable from the control (Fig. 2B−D). In contrast to other groups, pSTAT1(Ser727) levels in the Senexin B group do not increase in response to M1 induction (Fig. 4A,C), possibly leading to the observed reduction in the expression of inflammatory response genes, such as IL-1β and Tnf (Fig. 2B,D). These findings suggest that in the Senexin B group, in the presence of CDK8/19 with inhibited kinase activity, the compensatory mechanisms of STAT1 phosphorylation by other kinases are disrupted, while compensation is possible in the case of complete absence of Cdk8/19 in the double knockout group.

The experimental results show that in Cdk19 and Cdk8/19 knockout macrophages, as well as in macrophages deprived of a CDK8/19 kinase function, inflammatory stimulation has different, sometimes opposite, effects on the expression of inflammation and glycolysis genes as well as activation of the key signaling pathways. Thus, next we aimed to assess how these differences affect surface markers characteristic of the M1 macrophage phenotype.

**Inhibition of CDK8/19 kinase activity reduces CD86 expression**

Next, we analyzed the effect of CDK8/19 deficit on the expression of surface marker CD86 under inflammatory stimulation. Differentiated macrophages were stimulated with IFN-γ and LPS for 24 h, followed by detachment from plastic and staining with anti-CD14 and anti-CD86 antibodies.

**Fig. 5:**
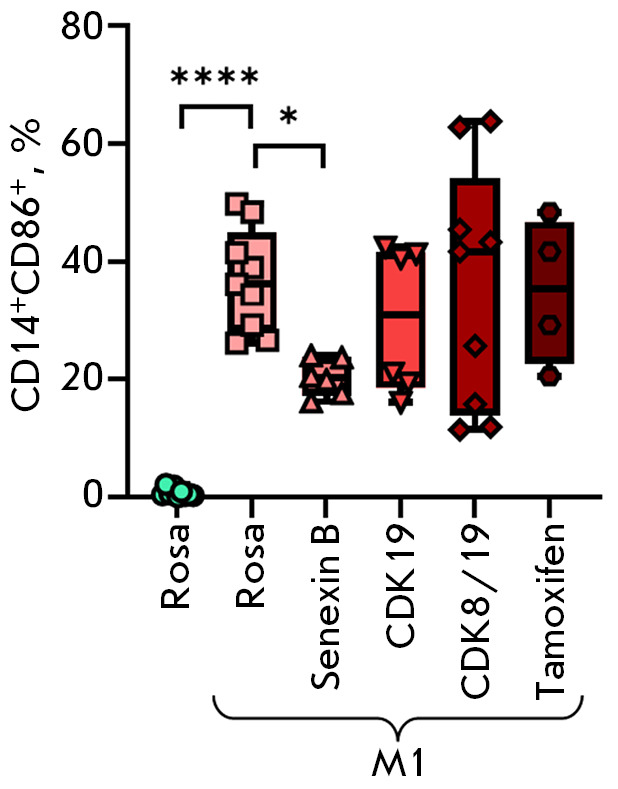
Analysis of macrophage surface markers. The values for stimulated experimental macrophages were compared to those obtained for stimulated Rosa group macrophages (*p < 0.05; ****p < 0.0001)

Interestingly, inhibition of CDK8/19 kinase activity is accompanied by a reduction in the expression levels of CD86, an M1 marker, while Cdk8/19 and Cdk19 knockouts do not have such an effect (Fig. 5). We suppose that under knockout conditions, CD86 activation is maintained by the activation of alternative pathways, which is impossible in the presence of CDK8/19 with inhibited kinase activity in the Senexin B group. That is consistent with the reduced activation of IL-1β and Tnf (Fig. 2B,D) in this experimental group.

**Cdk19 knockout promotes the reduction in lipid inclusions accumulation**

**Fig. 6:**
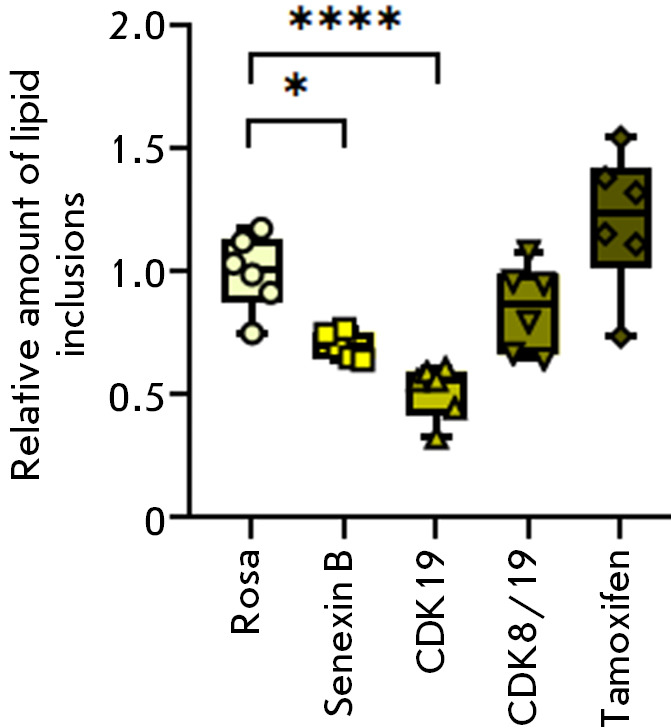
Assessment of lipid inclusions. The values for stimulated experimental macrophages were compared to those obtained for stimulated Rosa macrophages (*p < 0.05; ****p < 0.0001)

It has previously been shown that M1 macrophages stimulate de novo lipogenesis for immune system support. The increased content of lipid inclusions in macrophages is a result and characteristic of M1 polarization [37, 45, 46]. Since our previous experiments suggested that CDK8/19 kinases are potentially involved in the macrophage M1-to-M2 phenotype shift, we aimed to assess the lipid inclusions content in macrophages upon inflammatory stimulation. Our findings show that Cdk19 knockout and inhibition of CDK8/19 kinase activity significantly reduce the amount of lipid inclusions in macrophages upon inflammatory stimulation (Fig. 6).

The fact that Cdk19 knockout and CDK8/19 kinase activity inhibition reduce lipid accumulation, while Cdk8/19 double knockout has no effect, allows one to conclude that there exists a functional antagonism between CDK8 and CDK19 in lipid metabolism regulation. We speculate that CDK19 is a lipogenesis activator, and Cdk19 knockout disrupts the process. It is possible that CDK8 acts as a lipogenesis suppressor due to its non-kinase functions. In macrophages with Cdk8/19 double knockout, both the activator (CDK19) and the suppressor (CDK8) are eliminated, leading to reciprocal compensation for their effects and restoration of the initial levels of lipid inclusions. Moreover, only Cdk8/19 double knockout macrophages display reduced activation of Tpi1 upon inflammation stimulation (Fig. 3C). Accumulation of its substrate DHAP may generate a precursor pool for the synthesis of lipids (glycerol-3-phosphate), which also agrees with the observed lipid profile in this group [39].

**Cdk19 knockout reduces the phagocytic activity of macrophages**

**Fig. 7:**
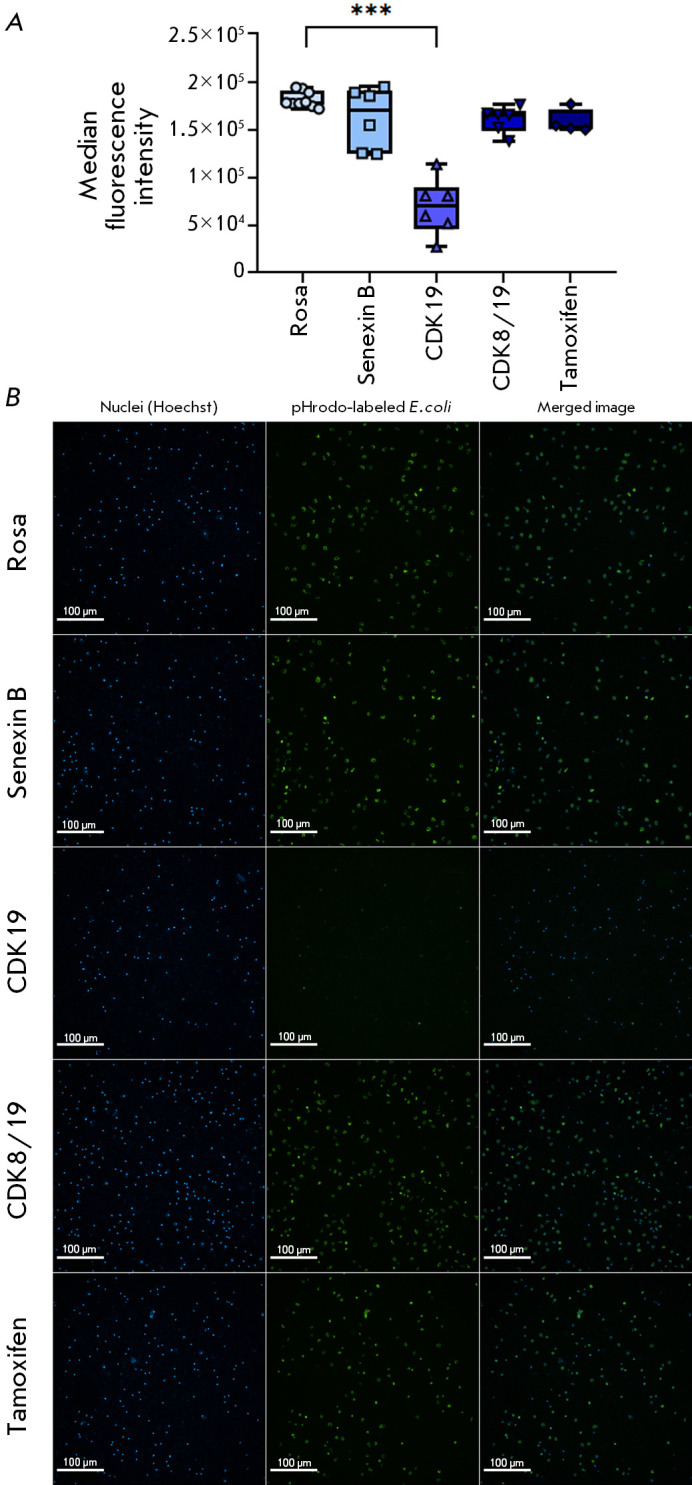
Assessment of the phagocytic activity of macrophages by (A) fluorescence flow cytometry and (B) fluorescent microscopy. The median fluorescence intensity in each experimental macrophage group was compared to that obtained for Rosa group macrophages (***p < 0.001)

Phagocytic activity [47] that ensures the elimination of bacteria and apoptotic cell fragments by M1 and M2 macrophages, respectively, is one of the main characteristics of macrophage. We have analyzed the ability to phagocytize pHrodo-labeled E. coli in Cdk19 and Cdk8/19 knockout macrophages, as well as in the Senexin B group. Our findings suggest that Cdk19 knockout significantly reduces the phagocytic activity when compared to the control (Fig. 7A,B). The observed reduction in phagocytic activity in Cdk19 knockout macrophages may be caused by the identified reduction in the lipid inclusions content. Phagocytosis is an energy-consuming process requiring resources for cytoskeleton rearrangements and phagosome formation; the reduction in internal lipid reserves depletes the cell’s necessary substrate and energy [48]. However, Cdk8/19 double knockout and kinase function inhibition did not affect phagocytosis, suggesting competitive antagonism between CDK8 and CDK19. Since we assumed that CDK8 acts as a lipogenesis suppressor, it stands to infer that its absence in Cdk8/19 double knockouts compensates for the disfunction caused by CDK19 deficit, restoring the normal levels of phagocytosis and lipid metabolism.

## CONCLUSIONS

Macrophage dysfunction is a key aspect of the pathogenesis of a number of socially significant human disorders associated with chronic inflammation, including atherosclerosis [1, 2] and rheumatoid arthritis [8, 16]. The M1 and M2 phenotypes of these cells largely determine the course and the outcome of the pathology, either aggravating or reducing the inflammation. Identification of the molecular mechanisms that control macrophage plasticity and polarization opens up opportunities for developing novel therapeutic strategies. In particular, induction of macrophage transition into the M2 state is considered a promising therapy approach. Cyclin-dependent kinase CDK8 and its paralog CDK19 are the components of the Mediator complex that regulates transcription in eukaryotes. These kinases have been shown to play a role in transcriptional reprogramming, a fundamental process underlying cell differentiation and the mechanisms of disease pathogenesis. Moreover, data on cytoplasmic CDK8/19 functions is available (e.g., on their role in signaling pathways regulation via phosphorylation of STAT proteins, as well as protein stability and degradation regulation). The cytoplasmic functions of CDK8/19 are less studied than the nuclear ones and are cell type-dependent [25, 49, 50]. Although their role in gene activation and repression has been well established, the fine mechanisms of CDK8/19- mediated transcriptional and cytoplasmic control are yet to be fully understood. In this work, we studied the role of CDK8/19 kinases in some aspects of the inflammation response in macrophages.

Our findings suggest that CDK8 and CDK19 not only regulate macrophage inflammatory response, but also modulate their intracellular metabolism and effector functions. CDK19 acts as a suppressor of the classical M1 response, although not via the STAT1- dependent mechanism. CDK19 inhibits activation of the IL-1β and Nos2 genes and, apparently, promotes lipid accumulation and phagocytosis. Cdk8/19 double knockout suppresses the macrophagic recruiting function and activates anti-inflammatory mediators, thus having a functional compensatory effect between two kinases. Importantly, inhibition of CDK8/19 kinase activity only partially recreates the observed effects (e.g., Tnf and IL-1β downregulation, reduced lipid accumulation), which suggests the presence of both kinase-dependent and kinase-independent mechanisms of action.

Our study demonstrates that the CDK8 and CDK19 kinases perform complex regulatory functions in macrophages that go beyond transcription control. The regulatory functions of these kinases are distinct and are implemented via both the kinase-dependent and kinase-independent mechanisms, thus regulating a wide range of processes: from gene expression to metabolism and phagocytosis. We hypothesize that in the absence of CDK19, CDK8 may become an activator of inflammatory response genes, which suggest there is competitive antagonism between CDK8 and CDK19 for common Mediator complex components. It is possible that the balance between them is responsible for the transcriptional response, although this hypothesis needs further study. Our findings emphasize that CDK8 and CDK19 are not just paralogs: they also act as regulators of transcription, the metabolic pathways, and effector functions of macrophages. Therefore, any therapeutic strategies targeting them should take this aspect into account.

## References

[1] Jacobs P., Bissonnette R., Guenther LC. (2011). Socioeconomic burden of immune-mediated inflammatory diseases - focusing on work productivity and disability.. J Rheumatol Suppl..

[2] Stone NJ. (1996). The clinical and economic significance of atherosclerosis.. Am J Med..

[3] Bobryshev YV., Ivanova EA., Chistiakov DA., Nikiforov NG., Orekhov AN. (2016). Macrophages and their role in atherosclerosis: pathophysiology and transcriptome analysis.. BioMed Res Int..

[4] Ajoolabady A., Pratico D., Lin L. (2024). Inflammation in atherosclerosis: pathophysiology and mechanisms.. Cell Death Dis..

[5] Chang JW., Tang CH. (2024). The role of macrophage polarization in rheumatoid arthritis and osteoarthritis: Pathogenesis and therapeutic strategies.. Int Immunopharmacol..

[6] Zheng Y., Wei K., Jiang P. (2024). Macrophage polarization in rheumatoid arthritis: signaling pathways, metabolic reprogramming, and crosstalk with synovial fibroblasts.. Front Immunol..

[7] Wu J., He S., Song Z. (2023). Macrophage polarization states in atherosclerosis.. Front Immunol..

[8] Theofilis P., Oikonomou E., Tsioufis K., Tousoulis D. (2023). The role of macrophages in atherosclerosis: pathophysiologic mechanisms and treatment considerations.. Int J Mol Sci..

[9] Liu T., Zhang L., Joo D., Sun SC. (2017). NF-κB signaling in inflammation.. Signal Transduct Target Ther..

[10] Xia T., Fu S., Yang R. (2023). Advances in the study of macrophage polarization in inflammatory immune skin diseases.. J Inflamm (Lond)..

[11] Zhao Y., Ma C., Chen C. (2022). STAT1 contributes to microglial/macrophage inflammation and neurological dysfunction in a mouse model of traumatic brain injury.. J Neurosci..

[12] Yamamoto S., Hagihara T., Horiuchi Y. (2017). Mediator cyclin-dependent kinases upregulate transcription of inflammatory genes in cooperation with NF-κB and C/EBP β on stimulation of Toll-like receptor 9.. Genes Cells..

[13] Liao X., Sharma N., Kapadia F. (2011). Krüppel-like factor 4 regulates macrophage polarization.. J Clin Invest..

[14] Rath M., Müller I., Kropf P., Closs EI., Munder M. (2014). Metabolism via arginase or nitric oxide synthase: two competing arginine pathways in macrophages.. Front Immunol..

[15] Yu T., Gan S., Zhu Q. (2019). Modulation of M2 macrophage polarization by the crosstalk between Stat6 and Trim24.. Nat Commun..

[16] Luo M., Zhao F., Cheng H., Su M., Wang Y. (2024). Macrophage polarization: an important role in inflammatory diseases.. Front Immunol..

[17] Javadifar A., Rastgoo S., Banach M., Jamialahmadi T., Johnston TP., Sahebkar A. (2021). Foam cells as therapeutic targets in atherosclerosis with a focus on the regulatory roles of non-coding RNAs.. Int J Mol Sci..

[18] Tian X., Chen J., Hong Y., Cao Y., Xiao J., Zhu Y. (2025). Exploring the role of macrophages and their associated structures in rheumatoid arthritis.. J Innate Immun..

[19] Hou P., Fang J., Liu Z. (2023). Macrophage polarization and metabolism in atherosclerosis.. Cell Death Dis..

[20] Dannappel MV., Sooraj D., Loh JJ., Firestein R. (2019). Molecular and in vivo functions of the CDK8 and CDK19 kinase modules.. Front Cell Dev Biol..

[21] Fant CB., Taatjes DJ. (2019). Regulatory functions of the Mediator kinases CDK8 and CDK19.. Transcription..

[22] Cozzolino KA., Sanford L., Hunter S. (2025). Mediator kinase inhibition suppresses hyperactive interferon signaling in Down syndrome.. elife..

[23] Dale T., Clarke PA., Esdar C. (2015). A selective chemical probe for exploring the role of CDK8 and CDK19 in human disease.. Nat Chem Biol..

[24] Liu Y., Sun L., Lu H. (2025). A comprehensive description of cyclin-dependent kinase 8 (CDK8) inhibitors as anticancer agents.. Bioorg Med Chem..

[25] Chen M., Li J., Zhang L. (2023). CDK8 and CDK19: positive regulators of signal-induced transcription and negative regulators of Mediator complex proteins.. Nucleic Acids Res..

[26] Guo Z., Wang G., Lv Y., Wan YY., Zheng J. (2019). Inhibition of Cdk8/Cdk19 activity promotes Treg cell differentiation and suppresses autoimmune diseases.. Front Immunol..

[27] Zarrin AA., Bao K., Lupardus P., Vucic D. (2021). Kinase inhibition in autoimmunity and inflammation.. Nat Rev Drug Discov..

[28] Li M., Hou Q., Zhong L., Zhao Y., Fu X. (2021). Macrophage related chronic inflammation in non-healing wounds.. Front Immunol..

[29] Hegarty LM., Jones GR., Bain CC. (2023). Macrophages in intestinal homeostasis and inflammatory bowel disease.. Nat Rev Gastroenterol Hepatol..

[30] Oishi Y., Manabe I. (2016). Macrophages in age-related chronic inflammatory diseases.. NPJ Aging Mech Dis..

[31] Bruter AV., Varlamova EA., Stavskaya NI. (2025). Knockout of cyclin-dependent kinases 8 and 19 leads to depletion of cyclin C and suppresses spermatogenesis and male fertility in mice.. elife..

[32] Mendoza R., Banerjee I., Manna D., Reghupaty SC., Yetirajam R., Sarkar D. (2022). Mouse bone marrow cell isolation and macrophage differentiation.. Methods Mol Biol..

[33] Liu L., Stokes JV., Tan W., Pruett SB. (2022). An optimized flow cytometry panel for classifying macrophage polarization.. J Immunol Methods..

[34] Šmerc A., Sodja E., Legiša M. (2011). Posttranslational modification of 6-phosphofructo-1-kinase as an important feature of cancer metabolism.. PLoS One..

[35] Jones W., Bianchi K. (2015). Aerobic glycolysis: beyond proliferation.. Front Immunol..

[36] Wang M., Zhou Q., Cao T. (2025). Lactate dehydrogenase A: a potential new target for tumor drug resistance intervention.. J Transl Med..

[37] Zhang C., Wang Y., Wang F. (2017). Quantitative profiling of glycerophospholipids during mouse and human macrophage differentiation using targeted mass spectrometry.. Sci Rep..

[38] Lu Y., Osis G., Zmijewska AA. (2025). Macrophage-specific lactate dehydrogenase expression modulates inflammatory function in vitro. . Kidney360..

[39] Chandel NS. (2021). Glycolysis.. Cold Spring Harb Perspect Biol..

[40] Orozco JM., Krawczyk PA., Scaria SM. (2020). Dihydroxyacetone phosphate signals glucose availability to mTORC1.. Nat Metab..

[41] Smith KR., Hayat F., Andrews JF., Migaud ME., Gassman NR. (2019). Dihydroxyacetone exposure alters NAD (P) H and induces mitochondrial stress and autophagy in HEK293T cells.. Chem Res Toxicol..

[42] Mehta R., Sonavane M., Migaud ME., Gassman NR. (2021). Exogenous exposure to dihydroxyacetone mimics high fructose induced oxidative stress and mitochondrial dysfunction.. Environ Mol Mutagen..

[43] Mallén-Ponce MJ., Quintero-Moreno AM., Gámez-Arcas S., Grossman AR., Pérez-Pérez ME., Crespo JL. (2025). Dihydroxyacetone phosphate generated in the chloroplast mediates the activation of TOR by CO2 and light.. Sci Adv..

[44] Neznamov AN., Baykova YP., Kubekina MV. (2026). The role of CDKs in the regulation of the monocyte/macrophage immune response.. Curr Med Chem..

[45] Batista-Gonzalez A., Vidal R., Criollo A., Carreño LJ. (2020). New insights on the role of lipid metabolism in the metabolic reprogramming of macrophages.. Front Immunol..

[46] Morgan PK., Huynh K., Pernes G. (2021). Macrophage polarization state affects lipid composition and the channeling of exogenous fatty acids into endogenous lipid pools.. J Biol Chem..

[47] Rosales C., Uribe-Querol E. (2017). Phagocytosis: a fundamental process in immunity.. Biomed Res Int..

[48] Chandak PG., Radović B., Aflaki E. (2010). Efficient phagocytosis requires triacylglycerol hydrolysis by adipose triglyceride lipase.. J Biol Chem..

[49] Szilagyi Z., Gustafsson CM. (2013). Emerging roles of Cdk8 in cell cycle control.. Biochimica et Biochim Biophys Acta..

[50] Audetat KA., Galbraith MD., Odell AT. (2017). A kinase-independent role for cyclin-dependent kinase 19 in p53 response.. Mol Cell Biol..

